# Effect of continuous statistically standardized measures of estrogen and progesterone receptors on disease-free survival in NCIC CTG MA.12 Trial and BC Cohort

**DOI:** 10.1186/bcr3465

**Published:** 2013-08-23

**Authors:** Judith-Anne W Chapman, Torsten O Nielsen, Matthew J Ellis, Phillip Bernard, Stephen Chia, Karen A Gelmon, Kathleen I Pritchard, Aurelie Le Maitre, Paul E Goss, Samuel Leung, Lois E Shepherd, Vivien H C Bramwell

**Affiliations:** 1 NCIC Clinical Trials Group, Queen's University, Kingston, ON K7L3N6, Canada; 2 Pathology Department, Vancouver Hospital, University of British Columbia, Vancouver, BC V5Z1M9, Canada; 3 Division of Medical Oncology, Washington University School of Medicine, St. Louis, MO 63110, USA; 4 Department of Pathology, University of Utah, Salt Lake City, UT 84112, USA; 5 Division of Medical Oncology, BCCA - Vancouver Centre, Vancouver, BC V5Z4E6, Canada; 6 Sunnybrook Odette Cancer Centre, University of Toronto, Toronto, ON M4N3M5, Canada; 7 Massachusetts General Hospital, Boston, MA 02114, USA; 8 Department of Oncology, University of Calgary, Calgary, AB T2N4N2, Canada

## Abstract

**Introduction:**

We hypothesized improved inter-laboratory comparability of estrogen receptor (ER) and progesterone receptor (PgR) across different assay methodologies with adjunctive statistical standardization, akin to bone mineral density (BMD) z-scores. We examined statistical standardization in MA.12, a placebo-controlled pre-menopausal trial of adjuvant tamoxifen with locally assessed hormone receptor +/- tumours, and in a cohort of post-menopausal British Columbia (BC) tamoxifen-treated patients.

**Methods:**

ER and PgR were centrally assessed for both patient groups with real time quantitative reverse transcription polymerase chain reaction (qPCR) and immunohistochemistry (IHC). Effects on disease-free survival (DFS) were investigated separately for 345 MA.12 and 673 BC patients who had both qPCR and IHC assessments. Comparisons utilized continuous laboratory units and statistically standardized z-scores. Univariate categorization of ER/PgR was by number of standard deviations (SD) above or below the mean (z-score ≥1.0 SD below mean; z-score <1.0 SD below mean; z-score ≤1.0 SD above mean; z-score >1.0 SD above mean). Exploratory multivariate examinations utilized step-wise Cox regression.

**Results:**

Median follow-up for MA.12 was 9.7 years; for BC patients, 11.8 years. For MA.12, 101 of 345 (29%) patients were IHC ER-PgR-. ER was not univariately associated with DFS (qPCR, *P *= 0.19; IHC, *P *= 0.08), while PgR was (qPCR, *P *= 0.09; IHC, *P *= 0.04). For BC patients, neither receptor was univariately associated with DFS: for ER, PCR, *P *= 0.36, IHC, *P *= 0.24; while for PgR, qPCR, *P *= 0.17, IHC, *P *= 0.31. Multivariately, MA.12 patients randomized to tamoxifen had significantly better DFS (*P *= 0.002 to 0.005) than placebo. Meanwhile, jointly ER and PgR were not associated with DFS whether assessed by qPCR or by IHC in all patients, or in the subgroup of patients with IHC positive stain, for pooled or separate treatment arms. Different results by type of continuous unit supported the concept of ER level being relevant for medical decision-making. For postmenopausal BC tamoxifen patients, higher qPCR PgR was weakly associated with better DFS (*P *= 0.06).

**Conclusions:**

MA.12 pre-menopausal patients in a placebo-controlled tamoxifen trial had similar multivariate prognostic effects with statistically standardized hormone receptors when tumours were assayed by qPCR or IHC, for hormone receptor +/- and + tumours. The BC post-menopausal tamoxifen cohort did not exhibit a significant prognostic association of ER or PgR with DFS. Adjunctive statistical standardization is currently under investigation in other NCIC CTG endocrine trials.

## Introduction

The growth of many breast cancers is hormone-dependent, with estrogen receptor (ER) and/or progesterone receptor (PgR) expression a prerequisite for responsiveness to endocrine therapy. Increased awareness about uncertainties in accurate assessment of these pivotal breast cancer biomarkers has renewed interest in standardization; there is the potential that 20% of current immunohistochemical (IHC) assay results worldwide are either false negatives or false positives [1]. Aspects affecting assays include tumor heterogeneity, acquisition and processing of specimens, antibody choices, laboratory assessment protocols, reproducibility of procedures, external assessment of process, proficiency of laboratory workers, sufficiency of scoring positivity and cut-points for positivity [1]. The American Society of Clinical Oncology and the College of American Pathologists (ASCO/CAP) recently published guideline recommendations for IHC testing of ER and PgR in breast cancer [1]. The Panel recommended a cut-off of a minimum of 1% of tumor cells positive for ER/PgR for a specimen to be considered positive [1].

Chia *et al. *[2] centrally assessed ER and PgR in the NCIC Clinical Trials Group Breast Committee Mammary (MA).12 (NCIC CTG MA.12) placebo-controlled trial of tamoxifen in premenopausal women; they utilized the new 1% cut-off for IHC positivity to examine the prognostic and predictive associations of ER and PgR with relapse-free and overall survival. Neither hormone receptor was found to be prognostic or predictive. However, intrinsic subtyping by PAM50 was prognostic and luminal subtypes were predictive of benefit from tamoxifen.

Welsh *et al*. focused standardization of ER assessments on the determination of ER positivity using automated quantitative immunofluorescence (QIF) [3] which has a broader range of detection than IHC, possibly minimizing false negative results. Cell lines with ER immunoreactivity were analyzed with QIF for standardization reliant on threshold intensity. Cut-offs at 10% or 1% did not greatly alter the proportion of positive tumours [3]. Further, Iwamoto *et al*. found that the small number of patients with 1% to 9% positive tumours is molecularly similar to ER-positive patients [4].

Bartlett *et al. *[5] investigated the role of continuous ER and PgR with the Tamoxifen and Exemestane Adjuvant Multinational (TEAM) trial data. They found significant prognostic effects with increasing values of continuous ER and PgR associated with higher disease-free survival (DFS) in the short (maximum 2.75 years) follow-up period before tamoxifen patients switched to exemestane [5].

We hypothesized that the process of statistical standardization originally envisaged to improve inter-laboratory comparability of ER/PgR assay results might be useful to improve comparability of results between assay methods. We investigate here the association of continuous ER and PgR with DFS in patients randomized to tamoxifen or placebo regardless of locally determined ER and PgR tumour status. Central review permitted investigation of statistical standardization for IHC and qPCR assessment modalities [6-10] across a broad range of hormone receptor values.

## Methods

### Patients

#### NCIC CTG MA.12

NCIC CTG MA.12 was a placebo-controlled trial of tamoxifen therapy following adjuvant chemotherapy in premenopausal women with early breast cancer [11] [see Additional file 1 CONSORT Diagram]. The study was approved by local research Ethics Boards, and patients provided written informed consent [11]. The NCIC CTG MA.12 Study Chair (VHCB), Physician Coordinator (LS) and sources of qPCR and IHC hormone receptor data (TON, SC, PB, MJE) gave permission to use MA.12 data in this work.

Patients with pathological T1-4, N0-2, M0 tumours were eligible. Local centre determination of levels of at least one hormone receptor (ER and/or PgR), by biochemical (positive ≥10 fmol/mg protein) or immunohistochemical assay was required, but patients with any receptor status were eligible. The stratification factors were type of chemotherapy (cyclophosphamide, methotrexate and fluorouracil (CMF); cyclophosphamide, epirubicin, fluorouracil (CEF); doxorubicin (adriamycin)/cyclophosphamide (AC)), hormone receptor status (ER and/or PgR positive, ER and PgR negative) and nodal status (0, 1 to 3, 4 to 9, 10+). The primary endpoint was overall survival (OS). DFS was a secondary endpoint and was defined as being the time from randomization to the earliest date of recurrence or death; censoring was the last date the patient was known to be alive.

A total of 672 women were accrued to MA.12, 338 randomized to tamoxifen and 334 to placebo. Tumour hormone receptor status was positive in 505 (75%) of women. At 9.7 years median follow-up, multivariate analysis showed a DFS benefit for tamoxifen of borderline significance (*P *= 0.056) and a trend for improved OS (*P *= 0.12). There was no evidence of greater efficacy for tamoxifen in the hormone-receptor positive or ER receptor-positive subgroups than in hormone-receptor negative or ER receptor-negative patients: interaction test *P *-values were, respectively, 0.71 and 0.14.

The process of statistical standardization requires continuous assay assessments, assessed by the same assessment method, in the same laboratory, under similar circumstances, for a sufficient number of patients to characterize the assay results with a normal distribution. The 672 MA.12 patients were accrued at 44 Canadian centres, with multiple different laboratories assaying tumours for hormone receptor status. Further, many of the patients entered MA.12 following biochemical assay of ER/PgR or with IHC results categorized as positive or negative. Thus, the local hormone receptor data were not suitable for our investigations. MA.12 patients with ER and PgR centrally assessed by qPCR and IHC did not apparently differ in baseline characteristics from all patients randomized to the trial [2].

#### British Columbia patient cohort

Adjuvant endocrine therapy would currently be considered for patients assessed to have hormone receptor positive tumours, regardless of menopausal status. Our investigations were augmented here with a cohort of 767 British Columbia breast cancer patients [12] who had central assessment of ER and PgR in the same laboratory as the MA.12 patients. The BC patients were all women with new primary breast cancer, who received adjuvant tamoxifen, without adjuvant chemotherapy. Only 22 of the patients were pre-menopausal, and 11 had unknown menopausal status, so we restricted investigations to the post-menopausal patient group. We defined a MA.12 DFS endpoint for the BC patients as time from randomization to the earliest date of recurrence or death, censoring at the last date the patient was known to be alive, or if alive, at June 30, 2004.

### Central review

ER and PgR were centrally assessed in the laboratory of TN by real time quantitative RT-PCR (qRT-PCR) and by IHC. Following pathologist review of formalin-fixed, paraffin-embedded source blocks stored at the NCIC-CTG Pathology office, two 0.6 mm cores were removed from representative areas of viable invasive carcinoma for tissue microarray construction, and two 1.0 mm cores were removed for RNA purification and qPCR determination of ER (*ESR1*) and PR (*PGR*) using the PAM50 assay method [12]. IHC analyses were performed on 4-micron sections from the tissue microarray, with ER assessed using ASCO/CAP compatible methods [1] (MA.12 trial: SP 1 rabbit monoclonal antibody (ThermoFisher Scientific, Fremont, CA, USA), using 1:50 dilution for 32 minutes with heat, and mild CC1 on Ventana BenchMark. PgR was similarly assessed with rabbit monoclonal 1E2 (Ventana, Tuscon, AZ, USA), pre-diluted for eight minutes with heat, and standard CC1 antigen retrieval and incubation; BC cohort: 6F11 mouse monoclonal antibody (Leica Biosystems Newcastle Ltd, UK), using 1:50 dilution for two hours with no heat, and standard CC1 on Ventana Dixcovery XT)). ER and PgR IHC were assessed by a pathologist as a visual score from 0 to 100% based on the fraction of invasive cancer nuclei positive above background.

### Statistical methods

ER and PgR qPCR data were log_2 _transformed; laboratory ER and PR zeros were treated as missing. Meanwhile, for ER and PgR IHC% positive stain, the Box-Cox log_e _transformation was indicated for variance stabilization, after addition of 0.1 to IHC ER and PgR zeros to permit the transformation. For each hormone receptor assessment method, the continuous logarithmic values were converted to statistically standardized z-scores using the assessment method mean and SD of logarithmic values:

z- score = ((log value - mean of log values)÷SD of log values), which has approximately a standard normal distribution, N(0,1).

For comparability, DFS investigations included patients who had both ER and PgR assays, by both qPCR and IHC. With the MA.12 trial, we investigated the effects of ER and PgR for: 1) all women regardless of ER and PgR status, referred to hereafter as all patients; and 2) the subgroup of these patients with centrally confirmed positive IHC staining for ER and/or PgR tumours; for patients allocated to 1) placebo, 2) tamoxifen or 3) both arms together. All the BC postmenopausal patients received tamoxifen and were assessed as a single group.

DFS was the endpoint utilized here to investigate the association between ER and PgR and outcome. Univariate tests for MA.12 utilized the stratified log-rank statistic; for the BC cohort, we used the generalized Wilcoxon (Peto-Prentice) test statistic. Graphical description was with Kaplan-Meier plots. For MA.12, we plotted the experience for IHC ER/PR zero and for ER/PgR positive stain, while for the BC group all patients were IHC ER positive. Analogous to bone mineral density (BMD), we used cut-points for positive stain categorization of number of standard deviations (SD) above/below the mean (z-score ≥1.0 SD below mean; z-score <1.0 SD below mean; z-score ≤1.0 SD above mean; z-score >1.0 SD above mean).

Exploratory multivariate examinations were with adjusted Cox regression, stratified for MA.12 by the stratification factors of nodal status and chemotherapy type. We investigated the effects of ER and PgR in continuous laboratory and statistically standardized z-scores. To permit comparison across assessment methods, ER and PgR had forced inclusion in all models, while for MA.12 trial therapy and baseline patient characteristics (age, pathological stage, pathological T stage, ECOG performance) were added in step-wise mode (*P *≤0.05). Factors considered for the BC cohort were age, MA.12 categories for number of positive nodes and clinical T stage; none of the patients received adjuvant chemotherapy.

## Results

### MA.12 patients

**Figure 1:**
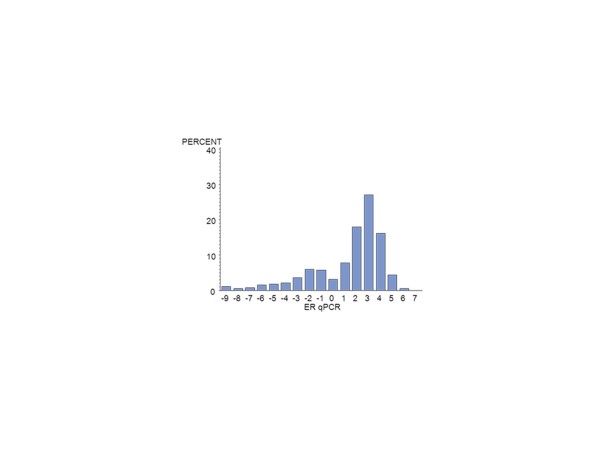
**Histogram of the NCIC CTG MA.12 qPCR (log_2_) ER assay results for all patients: N = 385**. ER, estrogen receptor.

**Figure 2:**
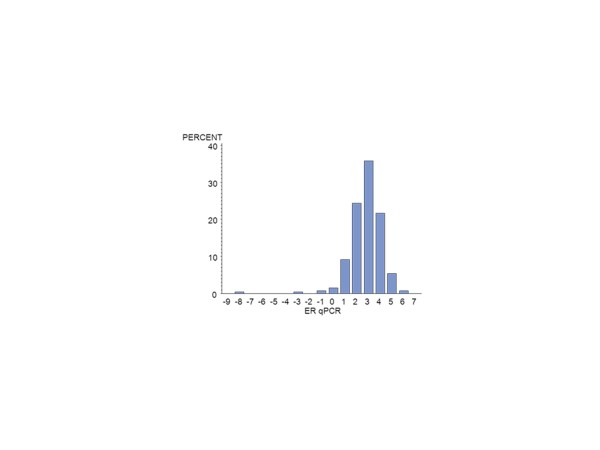
**Histogram of the NCIC CTG MA.12 qPCR (log_2_) ER results for central IHC ER and/or PgR >0: N = 263**. ER, estrogen receptor; IHC, immunohistochemistry; PgR, progesterone receptor.

**Figure 3:**
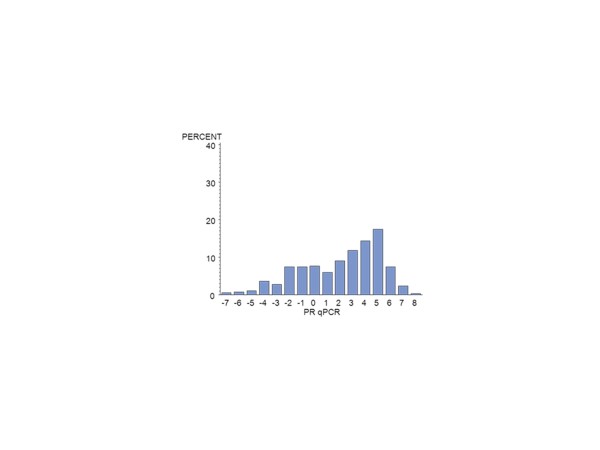
**Histogram of the NCIC CTG MA.12 qPCR (log_2_) PgR results for all patients: N = 389**. PgR, progesterone receptor.

**Figure 4:**
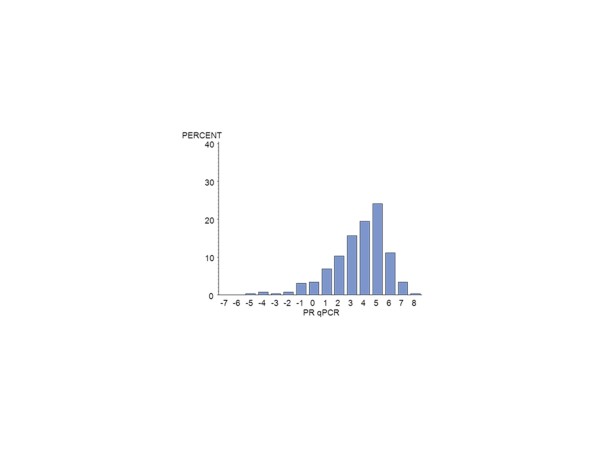
**Histogram of the NCIC CTG MA.12 qPCR (log_2_) PgR results for IHC ER and/or PgR >0: N = 262**. ER, estrogen receptor; IHC, immunohistochemistry; PgR, progesterone receptor.

Of the 672 MA.12 patients, centrally assessed ER was available by IHC for 392 (58%) and for PgR, for 376 (56%) patients. There were 124 centrally reviewed patients with no IHC staining for ER or PgR. Centrally assessed ER was available by qPCR for 385 (57%) patients and for PgR for 389 (58%). Figure 1 shows the qPCR ER assay results for all patients; Figure 2, the qPCR ER results for central IHC positive hormone receptor stain; Figure 3, the qPCR PgR results for all patients; and Figure 4, the qPCR PgR results for central IHC positive hormone receptor stain. Histograms of qPCR ER and PgR values in Figures 1 and 3 covered the spectrum of negative and positive IHC stain and exhibited bimodality. Meanwhile, qPCR values for tumours with only positive IHC stain in Figures 2 and 4 exhibit unimodal distributions. Corresponding IHC histograms are provided [see Additional files 2 to 5, Figures S1 to S4]; the best Box-Cox transformation was a logarithm, although the resulting IHC histograms do not indicate the same level of symmetry as those observed for qPCR.

**Figure 5:**
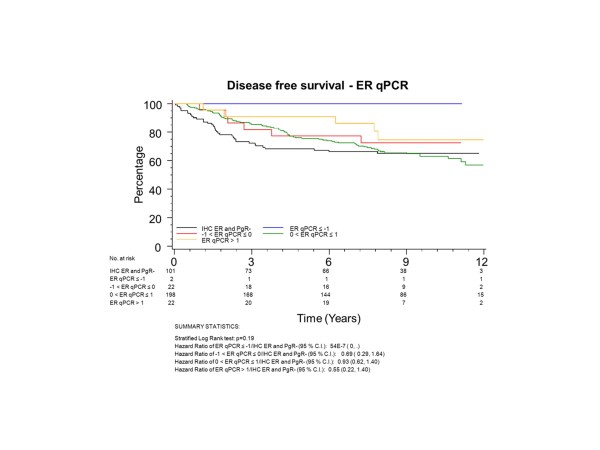
**NCIC CTG MA.12 Disease Free Survival by Standardized qPCR ER: **Assay results are categorized by their z-scores (greater to or equal to 1 standard deviation (SD) below mean, less than 1 SD below the mean, less than or equal to 1 SD above the mean, and greater than 1 SD above the mean) or whether tumors under central review had no IHC assay staining. ER, estrogen receptor; IHC, immunohistochemistry.

**Figure 6:**
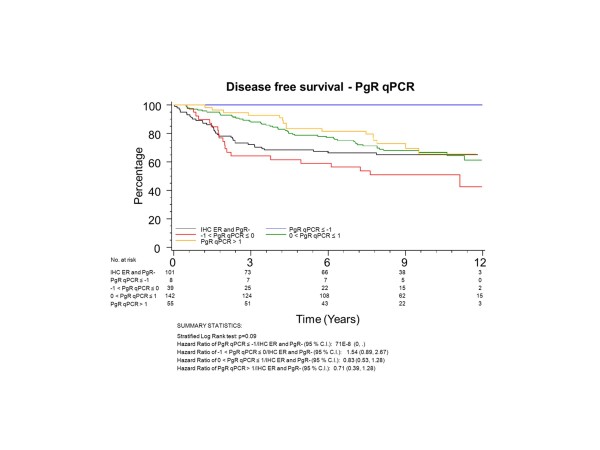
**NCIC CTG MA.12 Disease Free Survival by Standardized qPCR PgR: **Assay results are categorized by their z-scores (greater to or equal to 1 standard deviation (SD) below mean, less than 1 SD below the mean, less than or equal to 1 SD above the mean, and greater than 1 SD above the mean) or whether tumors under central review had no IHC assay staining. IHC, immunohistochemistry; PgR, progesterone receptor.

**Figure 7:**
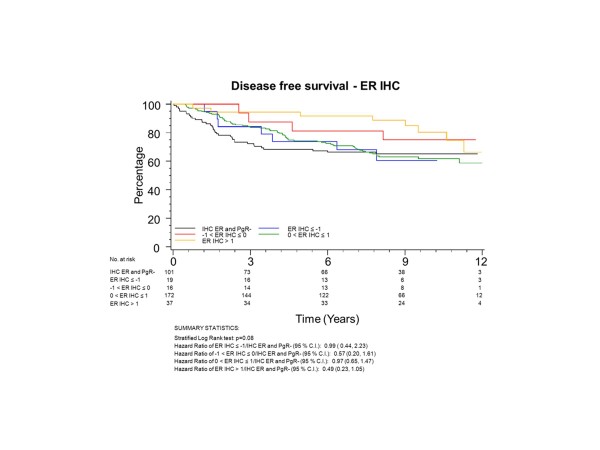
**NCIC CTG MA.12 Disease Free Survival by Standardized IHC ER: **Assay results are categorized by their z-scores (greater to or equal to 1 standard deviation (SD) below mean, less than 1 SD below the mean, less than or equal to 1 SD above the mean, and greater than 1 SD above the mean) or whether tumors under central review had no IHC assay staining. ER, estrogen receptor; IHC, immunohistochemistry.

**Figure 8:**
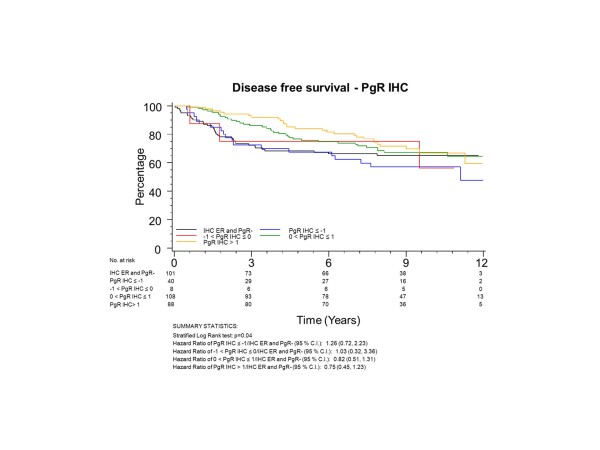
**NCIC CTG MA.12 Disease Free Survival by Standardized IHC PgR: **Assay results are categorized by their z-scores (greater to or equal to 1 standard deviation (SD) below mean, less than 1 SD below the mean, less than or equal to 1 SD above the mean, and greater than 1 SD above the mean) or whether tumors under central review had no IHC assay staining. IHC, immunohistochemistry; PgR, progesterone receptor.

To have the same patients included in comparisons across assessment methods, all further examinations were restricted to the group of 345 patients who had both ER and PgR assessed by both qPCR and IHC; 101 (29%) of these patients had tumours with no IHC stain for ER or PgR. The K-M plots (Figures 5 to 8) depict DFS experience for patients whose tumours under central review had no IHC ER or PgR staining, and DFS experience for ER or PgR assay results categorized by their Z-scores to be multiple SDs above or below the mean: greater to or equal to 1 SD below mean, less than 1SD below the mean, less than or equal to 1 SD above the mean, and greater than 1 SD above the mean. Univariately, qPCR ER was not associated with DFS (Figure 5, *P *= 0.19), while both qPCR PgR (Figure 6, *P *= 0.09) and IHC ER (Figure 7, *P *= 0.08) had weak evidence of association, and IHC PgR (Figure 8) achieved statistical significance (*P *= 0.04). There was a general indication that patients with ER and PgR staining z-score values >1.0, that is, >1.0 SD above the standardized mean, had better DFS, while those with no IHC ER and PgR stain had worse DFS.

Multivariate results are provided in Table 1. In all instances, patients randomized to tamoxifen had significantly better DFS (*P *= 0.002 to 0.005) than those allocated to placebo. However, patients randomized to the tamoxifen arm did not have significantly different DFS by ER or PgR levels, in continuous or standardized units, whether assessed by qPCR or IHC, in all patients, or in the subgroup of patients with IHC positive stain. Likewise, with pooling of patients on both treatment arms, there was no overall evidence for an association for ER or PgR with DFS. The single instance of standardized PgR being significant (*P *= 0.05) may easily be due to chance with the number of tests performed.

**Table 1:** NCIC CTG MA

	IHC ER *P*-value^1^	IHC PgR *P*-value^1^	qPCR ER *P*-value^1^	qPCR PgR *P*-value^1^	Therapy *P*-value^1^
I. Placebo Arm Cox Model for All Patients: All IHC values					

IHC	0.27	0.08	N/A	N/A	N/A

Standardized IHC	0.96	0.07	N/A	N/A	N/A

qPCR	N/A	N/A	0.12	0.02	N/A

Standardized qPCR	N/A	N/A	0.12	0.02	N/A

IHC and qPCR	0.08	0.41	0.03	0.15	N/A

Standardized IHC and qPCR	0.40	0.19	0.12	0.47	N/A

Cox Model for Patients with Positive IHC ER and/or PgR					

IHC	0.25	0.29	N/A	N/A	N/A

Standardized IHC	0.53	0.07	N/A	N/A	N/A

qPCR	N/A	N/A	0.23	0.01	N/A

Standardized qPCR	N/A	N/A	0.23	0.01	N/A

IHC and qPCR	0.01	0.67	0.04	0.01	N/A

Standardized IHC and qPCR	0.21	0.50	0.11	0.04	N/A

II. Tamoxifen Arm Cox Model for All Patients: All IHC values					

IHC	0.18	0.61	N/A	N/A	N/A

Standardized IHC	0.44	0.89	N/A	N/A	N/A

qPCR	N/A	N/A	0.46	0.83	N/A

Standardized qPCR	N/A	N/A	0.46	0.83	N/A

IHC and qPCR	0.26	0.69	0.79	0.95	N/A

Standardized IHC and qPCR	0.50	0.58	0.75	0.48	N/A

Cox Model for Patients with Positive IHC ER and/or PgR					

IHC	0.30	0.57	N/A	N/A	N/A

Standardized IHC	0.37	0.82	N/A	N/A	N/A

qPCR	N/A	N/A	0.22	0.19	N/A

Standardized qPCR	N/A	N/A	0.22	0.19	N/A

IHC and qPCR	0.75	0.57	0.34	0.20	N/A

Standardized IHC and qPCR	0.67	0.14	0.22	0.07	N/A

III. Both Arms Together Cox Model for All Patients: All IHC values					

IHC	0.20	0.45	N/A	N/A	0.003

Standardized IHC	0.63	0.21	N/A	N/A	0.004

qPCR	N/A	N/A	0.66	0.20	0.004

Standardized qPCR	N/A	N/A	0.45	0.19	0.003

IHC and qPCR	0.07	0.75	0.15	0.48	0.002

Standardized IHC and qPCR	0.28	0.22	0.20	0.99	0.003

Cox Model for Patients with Positive IHC ER and/or PgR					

IHC	0.18	0.43	N/A	N/A	0.004

Standardized IHC	0.47	0.05	N/A	N/A	0.005

qPCR	N/A	N/A	0.75	0.30	0.005

Standardized qPCR	N/A	N/A	0.75	0.30	0.005

IHC and qPCR	0.07	0.75	0.25	0.53	0.002

Standardized IHC and qPCR	0.34	0.10	0.55	0.97	0.005

^**1**^P-value is for factor's inclusion in stratified Cox model, with adjustment for stratification factors of nodal status and adjuvant chemotherapy.

There is inconsistent evidence of a prognostic effect for hormone receptors for patients randomized to the placebo arm. The evidence was strongest for qPCR PgR (*P *= 0.01 to 0.04 in three of four scenarios). The inconsistency is illustrated in two scenarios. For IHC hormone receptor positive and negative patients, laboratory value qPCR assessment alone indicated significant association of PgR with DFS (*P *= 0.02) while IHC alone indicated weak evidence for IHC PgR (*P *= 0.08). However, the joint consideration of laboratory IHC and qPCR assessments led to a qPCR PgR *P*-value of 0.15 (changed from 0.02) and IHC PgR *P*-value of 0.41 (changed from 0.08), with significant association for continuous qPCR ER (*P *= 0.03, changed from *P *= 0.12) and weak evidence for IHC ER (*P *= 0.08, changed from *P *= 0.27). Thus, there is a reversed indication of whether PgR or ER has the significant association with DFS.

The second example occurs in the subgroup with positive IHC hormone receptor stain. Laboratory qPCR assessment alone indicated PgR was significantly associated with DFS (*P *= 0.01), although in the joint consideration of IHC and qPCR, both qPCR ER (*P *= 0.04) and IHC ER (*P *= 0.01) were also significant. There was a change from only PgR being significant to both ER and PgR being significantly associated with DFS.

Further, substantive differences were noted on the placebo arm jointly considering both IHC and qPCR between ER assessed with or without standardization: for all patients regardless of IHC status, standardized qPCR ER *P *= 0.12 versus laboratory units *P *= 0.03; with positive IHC stain, standardized IHC ER, *P *= 0.21 versus laboratory units *P *= 0.01; standardized qPCR ER, *P *= 0.11 versus laboratory units *P *= 0.04. There are differences in results by type of continuous unit supporting the concept that level of ER beyond a dichotomous negative or positive stain could be relevant for medical decision-making.

### BC patients

**Figure 9:**
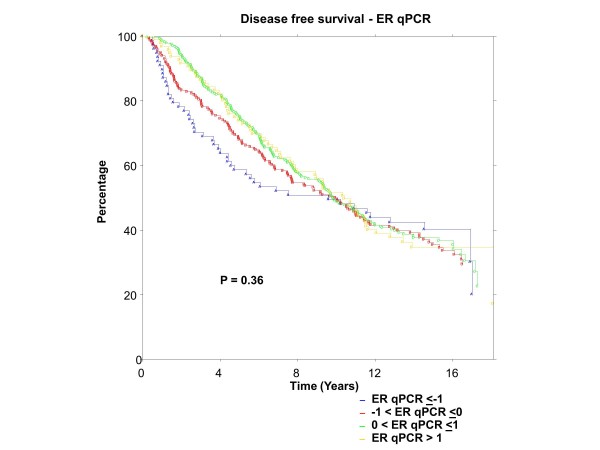
**BC Cohort Disease Free Survival by Standardized qPCR ER: **Assay results are categorized by their z-scores (greater to or equal to 1 standard deviation (SD) below mean, less than 1 SD below the mean, less than or equal to 1 SD above the mean, and greater than 1 SD above the mean). *P*-value is that for the generalized Wilcoxon (Peto-Prentice) test statistic. ER, estrogen receptor.

**Figure 10:**
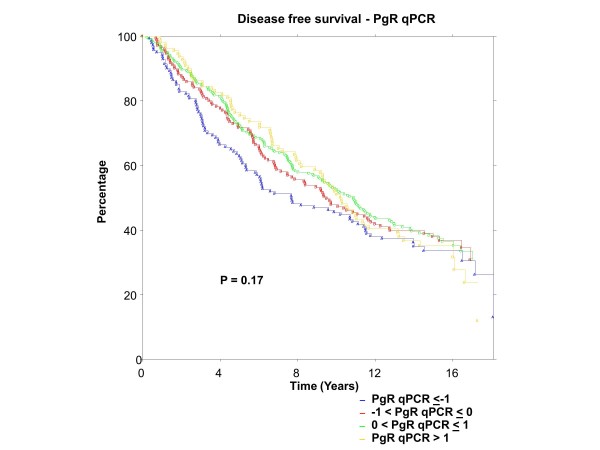
**BC Cohort Disease Free Survival by Standardized qPCR PgR: **Assay results are categorized by their z-scores (greater to or equal to 1 standard deviation (SD) below mean, less than 1 SD below the mean, less than or equal to 1 SD above the mean, and greater than 1 SD above the mean). *P*-value is that for the generalized Wilcoxon (Peto-Prentice) test statistic. PgR, progesterone receptor.

**Figure 11:**
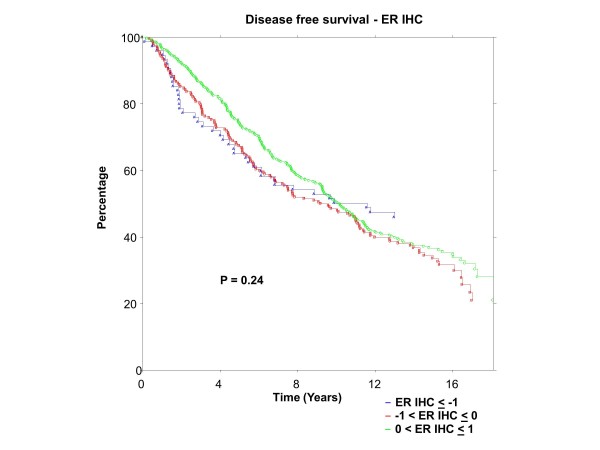
**BC Cohort Disease Free Survival by Standardized IHC ER: **Assay results are categorized by their z-scores (greater to or equal to 1 standard deviation (SD) below mean, less than 1 SD below the mean, less than or equal to 1 SD above the mean, and greater than 1 SD above the mean). *P*-value is that for the generalized Wilcoxon (Peto-Prentice) test statistic. ER, estrogen receptor; IHC, immunohistochemistry.

**Figure 12:**
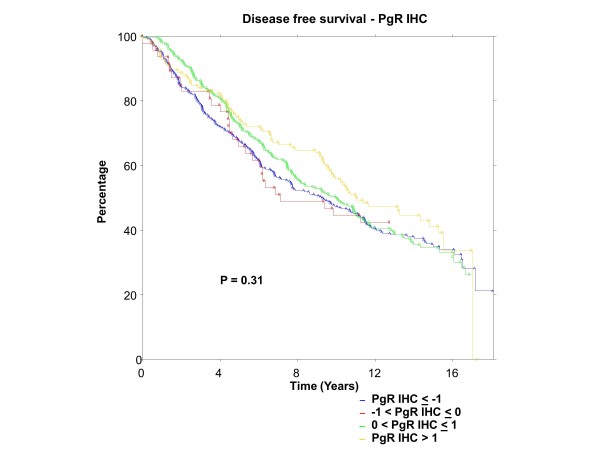
**BC Cohort Disease Free Survival by Standardized IHC PgR: **Assay results are categorized by their z-scores (greater to or equal to 1 standard deviation (SD) below mean, less than 1 SD below the mean, less than or equal to 1 SD above the mean, and greater than 1 SD above the mean). *P*-value is that for the generalized Wilcoxon (Peto-Prentice) test statistic. IHC, immunohistochemistry; PgR, progesterone receptor.

ER was centrally assessed by qPCR for 767 patients and IHC for 688 patients; PgR by qPCR for 767 patients and IHC for 717 patients. There were 673 of 767 (88%) patients who had central qPCR and IHC for ER and PgR. To have the same patients included in comparisons across assessment methods, all further examinations were restricted to this group of 673 patients, all of whom had IHC stain for ER and/or PgR. The K-M plots (Figures 9 to 12) depict DFS experience for patients whose tumours under central review had ER or PgR assay results categorized by their Z-scores to be multiple SDs above or below the mean: greater to or equal to 1 SD below mean, less than 1SD below the mean, less than or equal to 1 SD above the mean, and greater than 1 SD above the mean. Univariately, qPCR ER was not associated with DFS (Figure 9, *P *= 0.36), nor was qPCR PgR (Figure 10, P = 0.17), IHC ER (Figure 11, *P *= 0.24) or IHC PgR (Figure 12, *P *= 0.31). Similar to MA.12, there was a general indication that patients with ER and PgR staining z-score values >0., that is, those above the standardized mean had better DFS, while those with no IHC ER and PgR stain had worse DFS, although experience converged to being similar by about 10 years.

Multivariate results are provided in Table 2. All patients received adjuvant tamoxifen, without adjuvant chemotherapy. There was no evidence that ER was associated with DFS, and only weak multivariate evidence (*P *= 0.06) that higher PgR was associated with better DFS.

**Table 2:** BC POST-MENOPAUSAL COHORT: MULTIVARIATE EFFECTS OF ER, PgR, ON DFS

	IHC ER *P*-value^1^	IHC PgR *P*-value^1^	qPCR ER *P*-value^1^	qPCR PgR *P*-value^1^
IHC	0.76	0.31	N/A	N/A

Standardized IHC	0.76	0.31	N/A	N/A

qPCR	N/A	N/A	0.17	0.06

Standardized qPCR	N/A	N/A	0.17	0.06

IHC and qPCR	0.97	0.58	0.17	0.06

Standardized IHC and qPCR	0.97	0.58	0.17	0.06

^1^P-value is for factor's inclusion in Cox model, with adjustment for significant effects of age, number of positive nodes, and clinical T stage.

## Discussion

Breast cancer is a complex disease which displays both inter-case and intra-tumour heterogeneity [13-16]. Tumor ER and/or PgR positivity is a prerequisite for responsiveness to targeted therapy with an endocrine agent. Yet, inter-laboratory comparability of hormone receptor assay values is still problematic after decades of routine clinical assessment. Many laboratories do not participate in external quality assurance programs, and the use of a uniform method of assessment is not assured even for those that do [1,3,5,16-19]. Tumour levels of hormone receptors, ER α and PgR, and dynamic range of assessment methodology [3,20,21] impact indications for the presence of hormone receptors. Further, while markers such as HER2 are quite homogeneously expressed across a tumour, ER and, particularly, PgR [22] may be more heterogeneous. Finally, the current multitude of laboratory assessment methods, scoring and (prior to the recent ASCO/CAP Guideline recommendations^1^) dichotomous cut-points for positivity from 'any positivity' to an 'H-score of 50' [23] have been problematic [24-27].

Part of the controversy about ER and PgR cut-points for positivity has centred around the inability in most endocrine clinical trials to assess the effects of therapy in patients with false negative ER and PgR. NCIC CTG MA.12 had the unusual feature of patients being randomized to tamoxifen or placebo regardless of their locally determined ER and PgR, permitting an examination of the effects of endocrine therapy for the spectrum of hormone receptor values.

Central review of ER and PgR with both qPCR and IHC permitted a comparison of these two methods, as well as a demonstration of benefit with higher levels of ER and PgR positivity.

Lastly, statistical standardization within assessment methods provided a common set of z-scores which would be expected to improve inter-laboratory comparability.

The comparison in this work was across methodologic platforms since patients may now have ER and PgR assessed clinically in a variety of ways, with different intra-method variability as well as inter-laboratory variability by method. Differences for IHC alone were the subject of the ASCO/CAP guidelines^1^. PCR methods are more quantitative, producing continuous assay levels; however, there is a need to establish validity by level. We restricted investigations here, achieved in the same laboratory, to be for the same patients for both methods.

The methodology of categorizing ER- and PgR-positive stain by cut-points corresponding to z-score standard deviations (analogous to BMD studies) indicated general univariate support that high levels of hormone receptors led to better DFS, and no receptors to a worse outcome. IHC PgR was significantly (*P *= 0.04) associated with DFS while qPCR PgR (*P *= 0.09), qPCR ER (*P *= 0.19), and IHC ER (*P *= 0.08) were not.

In the current study, IHC analyses for ER and PgR had a stronger association with outcome than was seen with single gene measurements for *ESR1 *and *PGR*. Tamoxifen acts against the ER protein rather than its mRNA so perhaps this result is not surprising. One strength of qPCR over IHC is the ability to quantify multiple genes simultaneously as a signature, allowing a quantitative association of multi-gene expression with a luminal centroid that is a stronger predictor of endocrine therapy response than single gene measures (Chia SK *et al. *[2]). Here, we confined our study to single biomarkers and focused particularly on IHC, the primary diagnostic method used in current clinical practice.

In MA.12, we found inconsistent multivariate indications of prognostic effect for hormone receptors for patients allocated to the placebo arm. High correlations between ER and PgR likely influenced indications of significance; for example, for all patients, when qPCR and IHC were assessed separately, PgR significance was indicated, qPCR PgR (*P *= 0.02) and IHC PgR (0.08). Meanwhile, in joint consideration of the two assessment modalities, only qPCR ER (*P *= 0.03) was significantly prognostic.

Likewise, for patients with positive IHC stain, qPCR PgR (*P *= 0.01) was significant, although in joint examination we found qPCR PgR (*P *= 0.01) as well as qPCR ER (*P *= 0.04) and IHC ER (*P *= 0.01) to be significantly associated.

Previously, we saw indications that biochemical ER, or PgR, or both, were significantly associated with outcome [8]. The lack of consistent support for a single hormone receptor, or for a single assessment method, precludes focused application in clinical practice. Further, substantive differences were noted with or without statistical standardization of ER: respectively, qPCR ER, *P *= 0.12 versus 0.03; IHC ER, *P *= 0.21 versus 0.01; qPCR ER, *P *= 0.11 versus 0.04. Differences in results by type of continuous unit support the concept that level of ER beyond a dichotomous negative or positive stain is relevant for medical decision-making. Further, we suggest it is prudent at this time to consider that the conservative indications of significance with standardized units are appropriate. The literature is replete with transient indications of biomarker significance, such that the requirement for validation is now the norm.

We found that patients allocated to tamoxifen did not exhibit significant multivariate ER or PgR effects on DFS, nor were there significant hormone receptor effects when patient data on both tamoxifen and placebo arms were pooled. These results held for all patients and for those with positive IHC ER and/or PgR stain, for qPCR and IHC assessments, and with or without standardized units.

Chia *et al. *[2] did not observe differences in baseline characteristics between the main MA.12 trial population and those for whom there was central review of hormone receptors. In the main trial, there was weak evidence (*P *= 0.056) that tamoxifen improved DFS [11]. However, trial therapy in the centrally reviewed population was consistently associated with significant multivariate DFS (*P *= 0.002 to 0.005), in all centrally reviewed patients, and in the subgroup of women whose tumours had positive IHC stain for ER and/or PgR. The distribution of events in patients with centrally reviewed tumors was not representative of the full trial population.

Our work was broadened here with a BC cohort of postmenopausal patients, all of whom received adjuvant tamoxofen, without adjuvant chemotherapy. The qPCR and IHC assessments of ER and PgR were performed in the same laboratory (that of TON). As in the MA.12 trial, there was a general directional indication that univariate DFS was better with higher ER and PgR, although there appeared to be no difference after 10 years follow-up, and overall there was no significant effect of ER or PgR on DFS found by qPCR or IHC assay methods, with or without statistical standardization. ER and PgR did not exhibit significant multivariate effects on DFS, although there was weak evidence (*P *= 0.06) that patients with higher qPCR PgR had better DFS. We recognize the limitations in cohort data, that patient and tumour characteristics could have impacted clinical and patient decisions in treatment choice, affecting outcomes. We also recognize that the patient spectrum was reduced when only hormone receptor positive patients are considered, and there was a decision not to administer adjuvant chemotherapy.

However, we note that there is some commonality for this study as both MA.12 trial patients and the BC cohort had qPCR and IHC assay results assessed in the same laboratory. The juxtaposition of the MA.12 pre-menopausal trial where patients with locally determined hormone receptor positive and negative tumours were randomized to receive tamoxifen or placebo, with the BC postmenopausal patients who, with locally determined hormone receptor positive tumours, received tamoxifen extends the spectrum of patients, tumour characteristics, and experience. Both groups showed general univariate directions that higher ER and PgR were associated with better DFS. There was no multivariate evidence that ER and PgR had a significant prognostic effect on DFS for either study population.

Inter-laboratory comparability of ER assay results has been problematic for decades. A proposal in the early 1980s involved mathematical adjustment of laboratory assay values utilizing reference laboratory values, like the WHO mandated mathematical adjustment of prothrombin times. Meanwhile, a lack of inter-laboratory comparability for bone mineral density (BMD) was resolved for both research and clinical purposes by the WHO with mandated statistically standardized t-scores and z-scores based on routine comparisons with reference population values.

Work on the proposal for adjunctive statistical standardization of ER began with poor inter-laboratory comparability in provincial quality control samples in the late 1980s [6-10] with Ontario laboratories performing biochemical ER assessments using the dextran-coated charcoal radioligand method, continued after laboratories switched to the double monoclonal enzyme-immunoassay, ER-EIA [7], and eventually, to immunohistochemical assays [8,9]. We hypothesized improved inter-laboratory comparability of ER/PgR assay results with adjunctive statistical standardization, and we showed improved comparability with provincial quality control samples, and examined the process in cohort studies [6-10]. Continuous ER and PgR effects were indicated in time-to-event investigations with cohorts of breast cancer patients [8,9]. The routine clinical use of t-scores and z-scores for BMD demonstrates the feasibility of adjunctive statistical standardization of ER and PgR in breast cancer and suggests an approach that may be clinically useful for delineating significant predictive and prognostic effects of continuous ER and PgR at multiple standard deviations below or above the mean.

## Conclusions

The growth of many breast cancers is hormone-dependent, with estrogen receptor (ER) and/or progesterone receptor (PgR) expression a prerequisite for responsiveness to endocrine therapy. Increased awareness about uncertainties in accurate assessment of these pivotal breast cancer biomarkers has renewed interest in standardization; there is a potential that 20% of current IHC assay results worldwide are either false negatives or false positives [1].

We hypothesized that the process of statistical standardization, akin to bone mineral density (BMD) z-scores, and originally envisaged to improve inter-laboratory comparability of ER/PgR assay results, might be useful to improve comparability of results between qPCR and IHC assay methods. We demonstrated statistical standardization across assay methods in MA.12, a placebo-controlled trial of adjuvant tamoxifen in premenopausal women, with locally assessed hormone receptor +/- tumours. We saw evidence suggestive of an unspecified continuous prognostic effect for hormone receptors. This is the first clinical trial report about statistical standardization in the unique MA.12 trial to which premenopausal patients were accrued regardless of their locally determined hormone receptor status. Further, there was also directional evidence that BC postmenopausal patients receiving tamoxifen had better outcome in at least the first 10 years, when they have higher hormone receptor assay values.

A plethora of laboratory assessment methods are used to assess hormone receptors. We showed here in MA.12 that statistically standardized hormone receptors had similar multivariate prognostic effects on DFS when tumours were assayed by qPCR or by IHC, across a spectrum of hormone receptor +/- tumours. The BC cohort did not exhibit significant prognostic effects on DFS for ER or PR, by qPCR or by IHC, with or without statistical standardization. The process of statistical standardization would need to be laboratory specific, established iteratively and cumulatively against external quality assurance samples that cover the range of ER and PgR assay levels. We are examining statistical standardization in other NCIC CTG endocrine trials.

The process of statistical standardization is akin to BMD z-scores which are used in clinical practice, so it would be feasible to consider statistically standardizing hormone receptor assays.

## Abbreviations

ASCO/CAP, American Society of Clinical Oncology and the College of American Pathologists; BC, British Columbia; BMD, bone mineral density; DFS, disease-free survival; ER, estrogen receptor; IHC, immunohistochemistry; qPCR, quantitative reverse transcription polymerase chain reaction; PgR, progesterone receptor; SD, standard deviation; TEAM, Tamoxifen and Exemestane Adjuvant Multinational; WHO, World Health Organization.

## Competing interests

The authors declare that they have no competing interests.

## Authors' contributions

JWC conceived the idea for the study, designed the study, oversaw analyses and drafted the manuscript. TON completed the qPCR and IHC assessments, suggested use of the data for this purpose, and worked on drafting the manuscript. MEJ, PB and SC participated in acquisition of the qPCR and IHC assessments. KAG, KIP, PEG and LES contributed to the exposition of the work and drafting the manuscript. ALeM performed the analyses and contributed to the presentation of the work. VHCB participated in the development of the work in the context of the trial and worked on drafting the manuscript. SL assisted in the integration of the BC cohort data. All authors read and approved the final manuscript.

## Supplementary Material

Additional file 1 **NCIC CTG MA.12 CONSORT Diagram**. Click here for file

Additional file 2 **Figure S1**. Histogram of the NCIC CTG MA.12 IHC log (ER) assay results for all patients: N = 392. Click here for file

Additional file 3 **Figure S2**. Histogram of the NCIC CTG MA.12 IHC log (ER) results for central IHC ER and/or PgR>0: N = 266. Click here for file

Additional file 4 **Figure S3**. Histogram of the NCIC CTG MA.12 IHC log (PgR) results for all patients: N = 376. Click here for file

Additional file 5 **Figure S4**. Histogram of the NCIC CTG MA.12 IHC log (PgR) results for IHC ER and/or PgR>0: N = 262. Click here for file
