# Supplementary figures and images for: Effect of continuous statistically standardized measures of estrogen and progesterone receptors on disease-free survival in NCIC CTG MA.12 Trial and BC Cohort
Source: Breast Cancer Res. 2013 Aug 23;15(4):R71. doi: 10.1186/bcr3465 (PMC3978444; doi:10.1186/bcr3465)

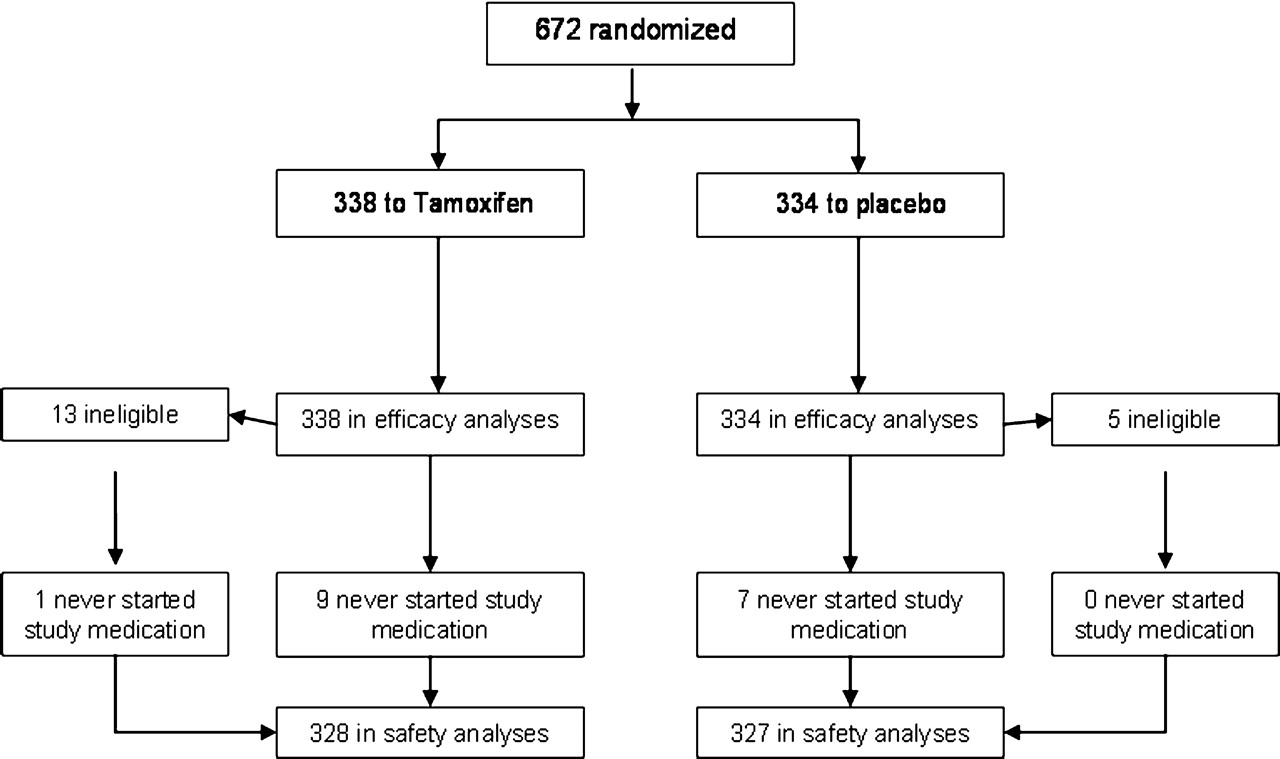

Supplement: Additional file 1 — NCIC CTG MA.12 CONSORT Diagram. [file bcr3465-S1.JPEG]

## Slide 1
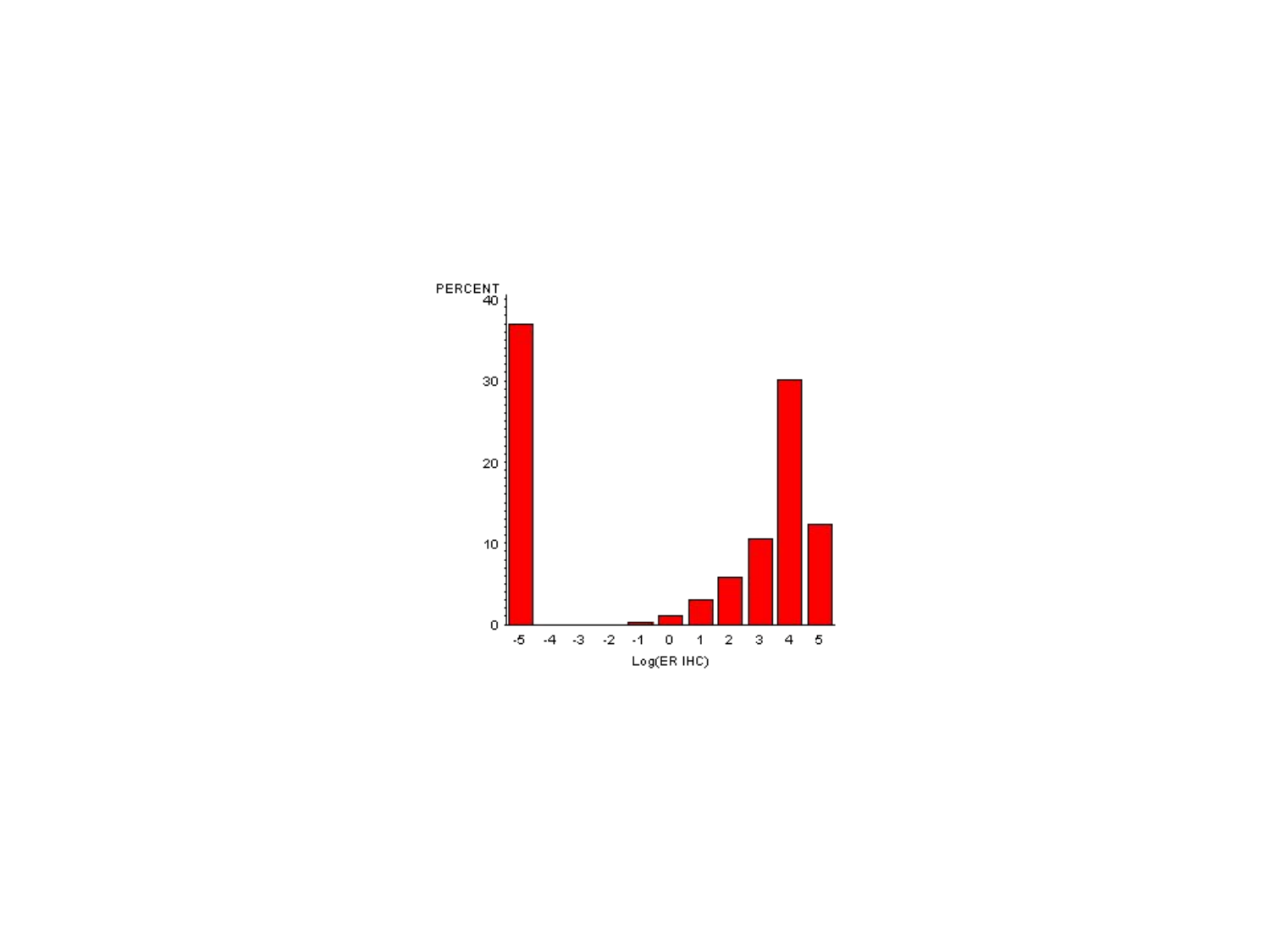

#

Supplement: Additional file 2 — Figure S1. Histogram of the NCIC CTG MA.12 IHC log (ER) assay results for all patients: N = 392. [file bcr3465-S2.PPTX]

## Slide 1
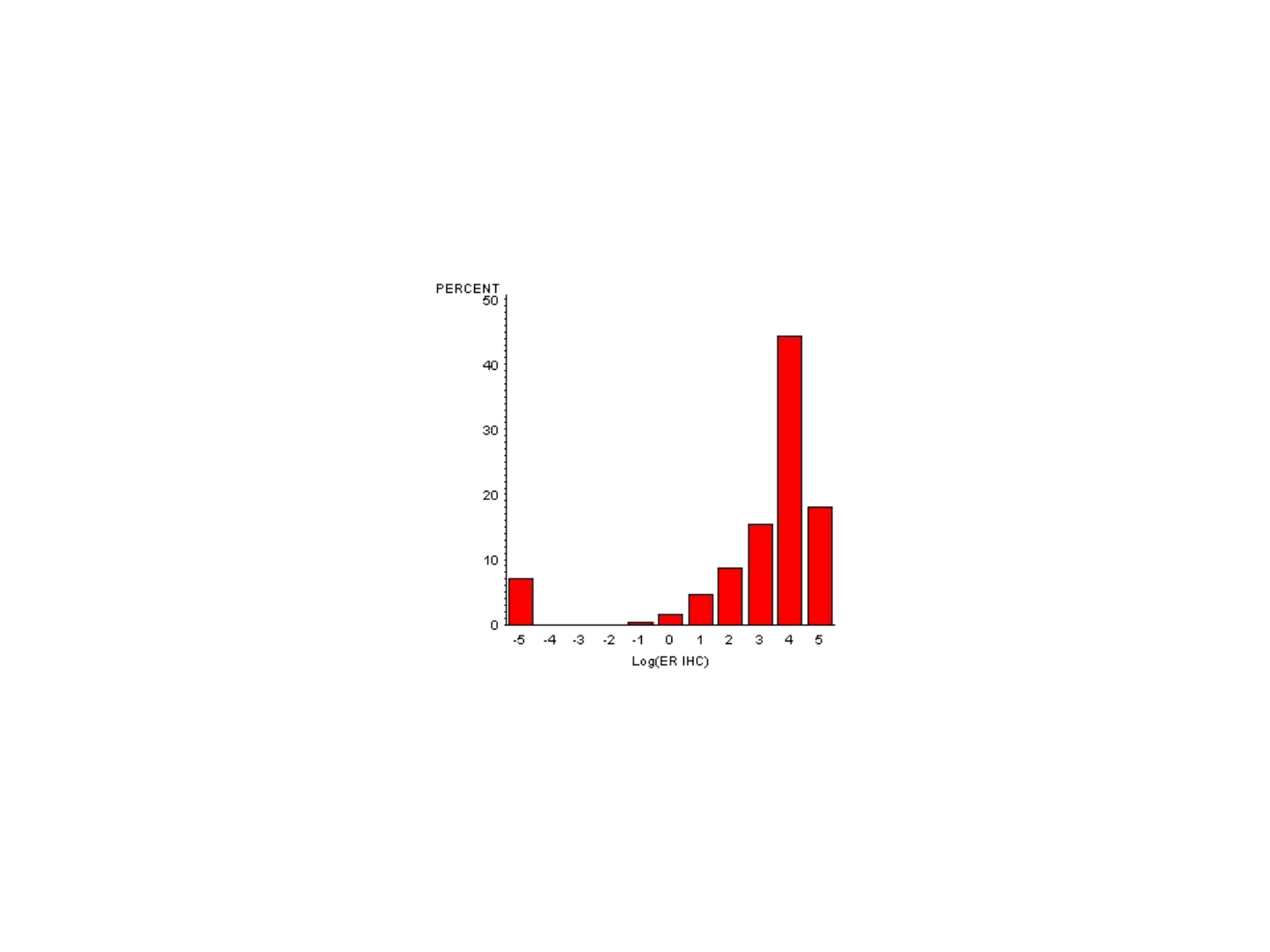

#

Supplement: Additional file 3 — Figure S2. Histogram of the NCIC CTG MA.12 IHC log (ER) results for central IHC ER and/or PgR>0: N = 266. [file bcr3465-S3.PPTX]

## Slide 1
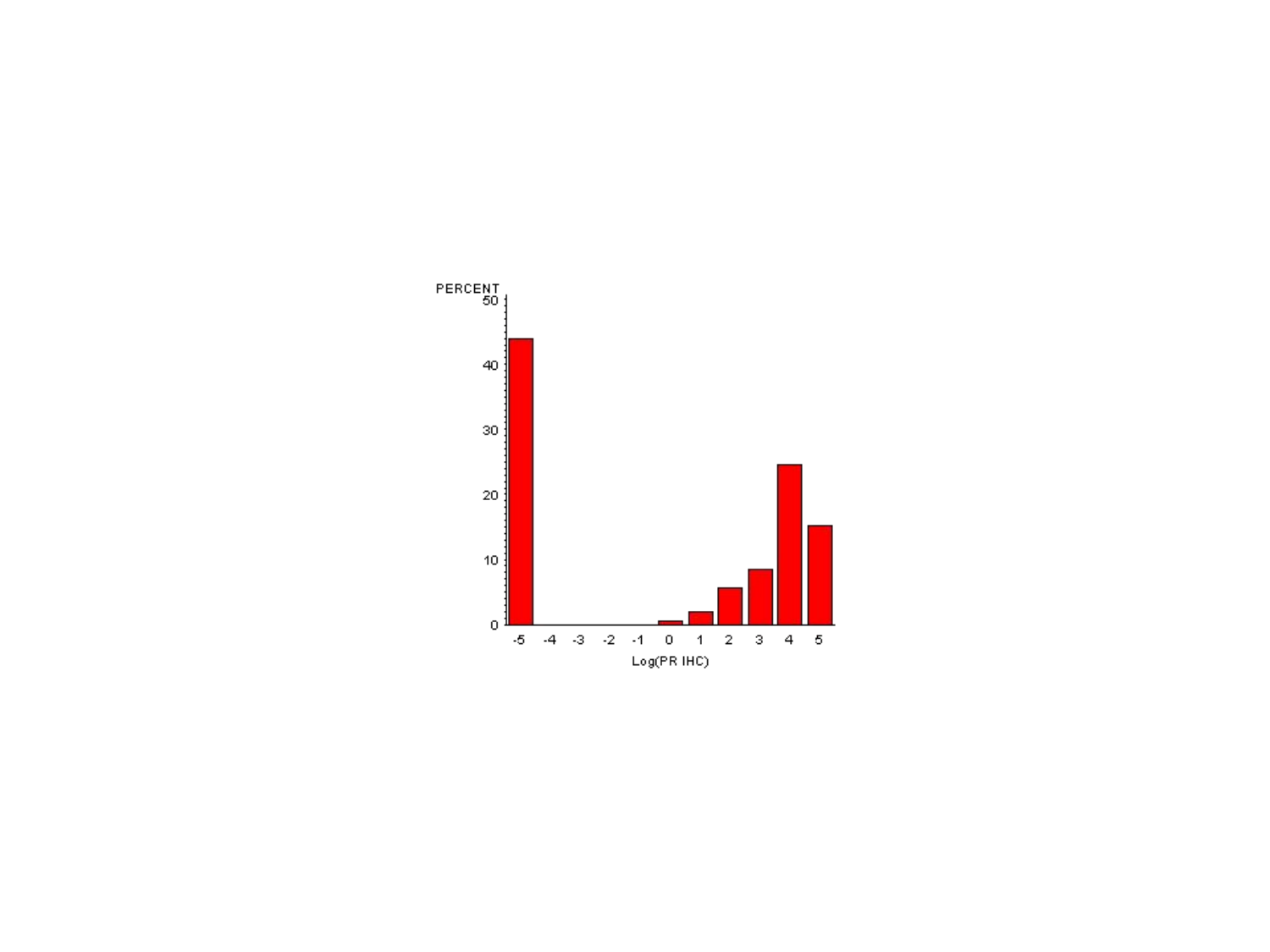

#

Supplement: Additional file 4 — Figure S3. Histogram of the NCIC CTG MA.12 IHC log (PgR) results for all patients: N = 376. [file bcr3465-S4.PPTX]

## Slide 1
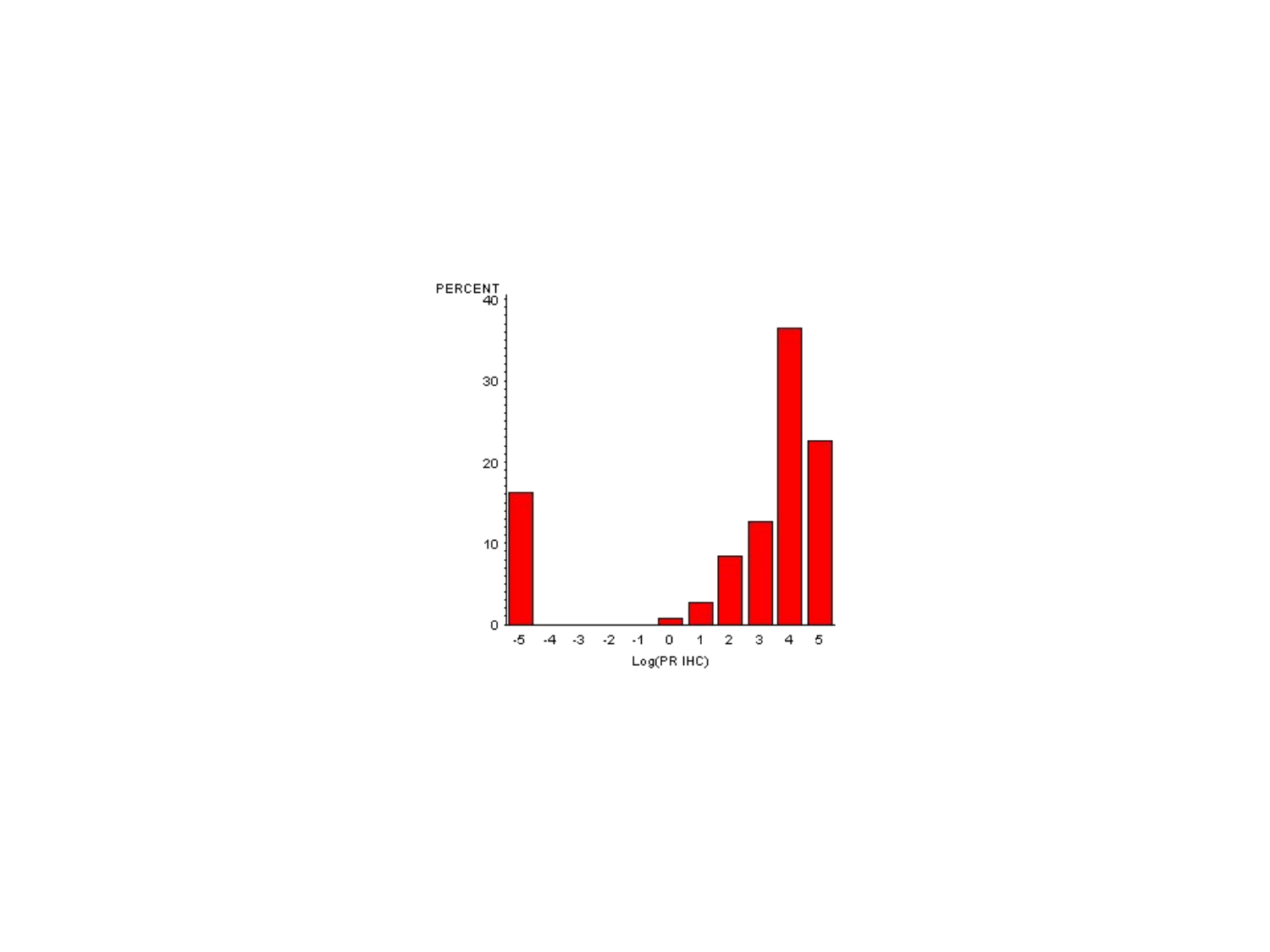

#

Supplement: Additional file 5 — Figure S4. Histogram of the NCIC CTG MA.12 IHC log (PgR) results for IHC ER and/or PgR>0: N = 262. [file bcr3465-S5.PPTX]
